# Supplementary figures and images for: A Macroscopic Mathematical Model for Cell Migration Assays Using a Real-Time Cell Analysis
Source: PLoS One. 2016 Sep 28;11(9):e0162553. doi: 10.1371/journal.pone.0162553 (PMC5040252; doi:10.1371/journal.pone.0162553)

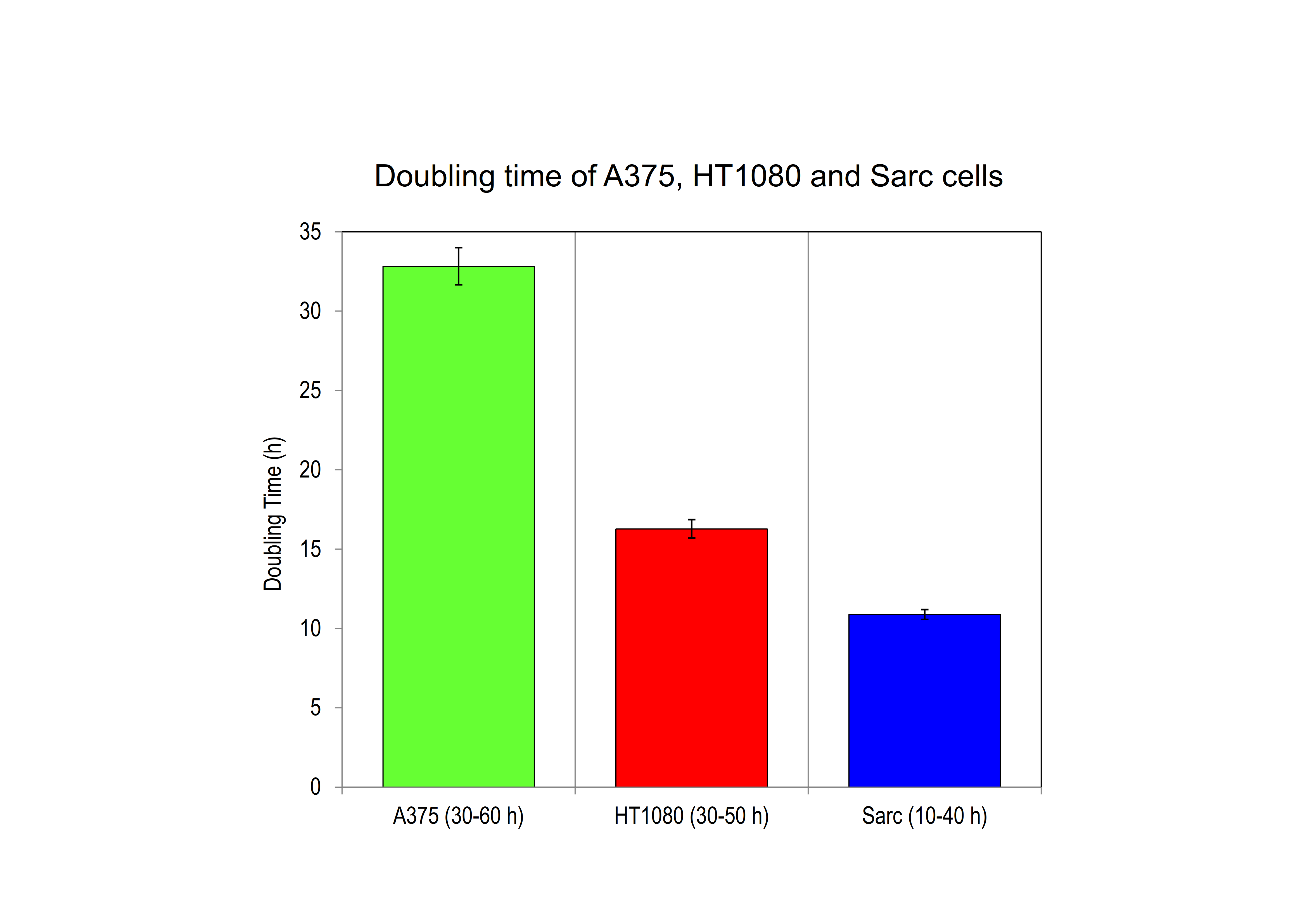

Supplement: S1 Fig — Cells (2 × 10^3^ cells/well) were seeded on E-plates and allowed to grow for 70 h in serum containing medium. The impedance value of each well was automatically monitored by the xCELLigence system and expressed as a Cell Index. Doubling times were calculated, using the xCELLigence RTCA software, from the cell growth curves during exponential growth given in round brackets for each cell line. Doubling time is expressed in term of mean value ± SD (standard deviation) from a quadruplicate experiment. (TIF) [file pone.0162553.s001.tif]

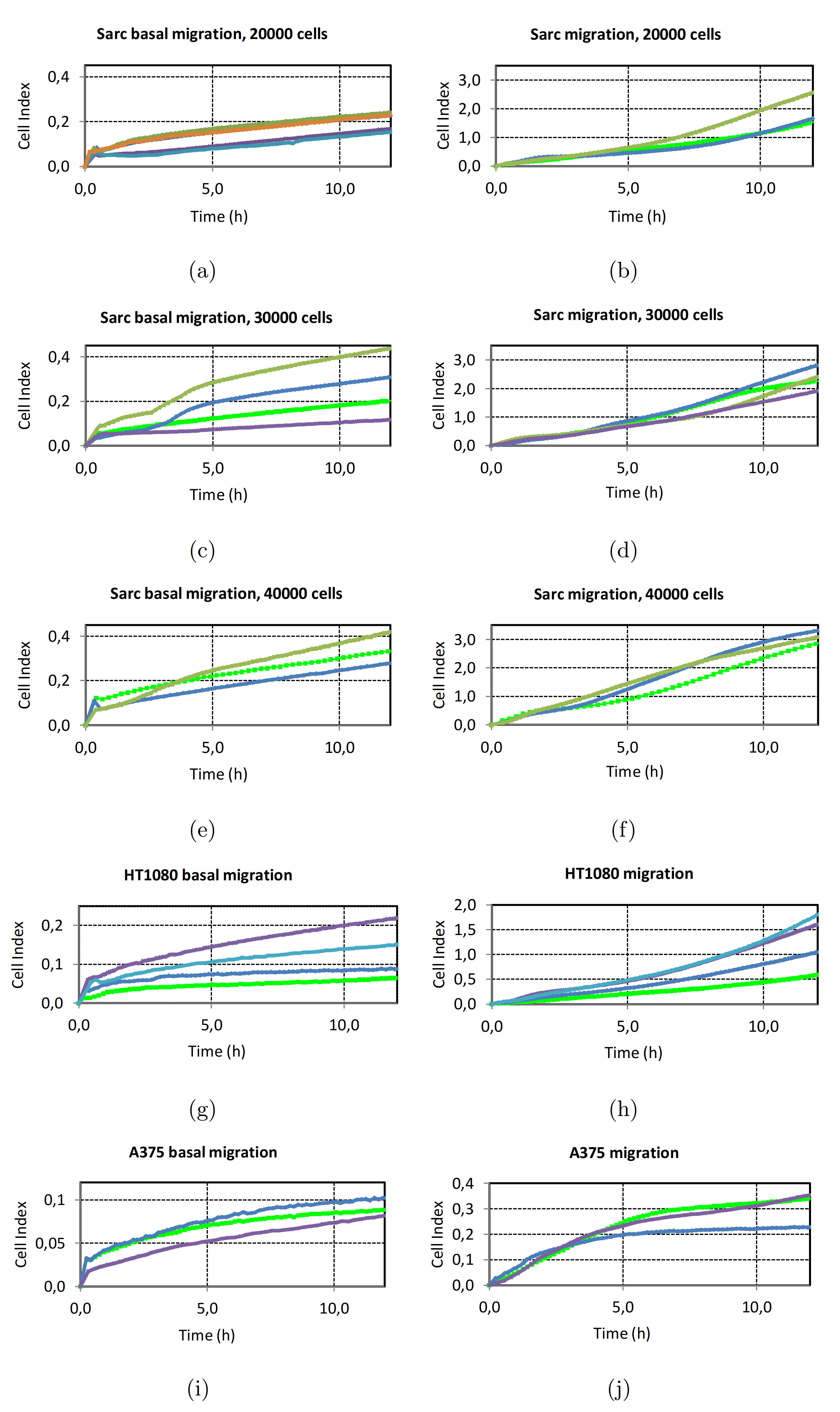

Supplement: S2 Fig — Panels (a),(c),(e),(g),(i) describe the basal migration, (b),(d),(f),(h),(j) the migration in presence of FBS. In each panel the curves represent an independent experiment carried out in quadruplicated and averaged. The observed curves in Figs 3 and 4 are obtained as the average of the curves showed here. (TIF) [file pone.0162553.s002.tif]
